# Signaling Pathway Reporter Screen with SARS-CoV-2 Proteins Identifies nsp5 as a Repressor of p53 Activity

**DOI:** 10.3390/v14051039

**Published:** 2022-05-13

**Authors:** Abhishek Kumar, Tristan R. Grams, David C. Bloom, Zsolt Toth

**Affiliations:** 1 Department of Oral Biology, University of Florida College of Dentistry, 1395 Center Drive, Gainesville, FL 32610, USA; akumar@dental.ufl.edu; 2 Department of Molecular Genetics and Microbiology, University of Florida College of Medicine, Gainesville, FL 32610, USA; tgrams@ufl.edu (T.R.G.); dbloom@ufl.edu (D.C.B.); 3 UF Genetics Institute, Gainesville, FL 32610, USA; 4 UF Health Cancer Center, Gainesville, FL 32610, USA

**Keywords:** SARS-CoV-2, COVID-19, coronavirus, nsp5, p53, signaling pathway

## Abstract

The dysregulation of host signaling pathways plays a critical role in severe acute respiratory syndrome coronavirus 2 (SARS-CoV-2) infection and viral pathogenesis. While a number of viral proteins that can block type I IFN signaling have been identified, a comprehensive analysis of SARS-CoV-2 proteins in the regulation of other signaling pathways that can be critical for viral infection and its pathophysiology is still lacking. Here, we screened the effect of 21 SARS-CoV-2 proteins on 10 different host signaling pathways, namely, Wnt, p53, TGFβ, c-Myc, Hypoxia, Hippo, AP-1, Notch, Oct4/Sox2, and NF-κB, using a luciferase reporter assay. As a result, we identified several SARS-CoV-2 proteins that could act as activators or inhibitors for distinct signaling pathways in the context of overexpression in HEK293T cells. We also provided evidence for p53 being an intrinsic host restriction factor of SARS-CoV-2. We found that the overexpression of p53 is capable of reducing virus production, while the main viral protease nsp5 can repress the transcriptional activity of p53, which depends on the protease function of nsp5. Taken together, our results provide a foundation for future studies, which can explore how the dysregulation of specific signaling pathways by SARS-CoV-2 proteins can control viral infection and pathogenesis.

## 1. Introduction

SARS-CoV-2, the causative agent of the coronavirus disease 2019 (COVID-19) pandemic, is an enveloped, 30 kb positive-sense single-stranded RNA virus that belongs to the genus Betacoronavirus in the family Coronaviridae [1,2]. The virus encodes sixteen non-structural proteins (nsp1-16) involved in the regulation of viral gene expression and replication, four structural proteins (S, E, M, and N), and a set of accessory proteins (orfs) that are not required for viral replication in vitro but are believed to play a role in pathogenesis [3,4]. Although SARS-CoV-2 preferentially infects cells in the respiratory tract, the virus has been reported to have a broad organotropism and can cause damage not only in the lungs but also, for example, in the heart, kidneys, liver, and brain [5,6]. While several vaccines have been developed against SARS-CoV-2, we still do not have any specific antiviral drugs that can halt the progression of the viral infection and its associated diseases [7]. The lack of effective antiviral therapies can be partly attributed to our insufficient knowledge about the basic biology of SARS-CoV-2 infection. This has prompted many laboratories to make significant efforts to identify host factors that are essential for SARS-CoV-2 infection and replication. So far, this has mainly been accomplished by using genome-wide functional genetic screens and virus–host protein interactome analyses, the goal of which is to identify proviral factors that can serve as targets for antiviral therapies against COVID-19 [8,9,10,11,12,13,14]. Although several animal models are being evaluated for studying the pathogenesis of SARS-CoV-2 infection, the majority of mechanistic studies on the functions of SARS-CoV-2 proteins have so far been performed in only a few epithelial cell lines [15].

Viruses are known to be able to effectively subvert immune response pathways and dysregulate cell signaling pathways to create a cellular milieu permissive for viral replication. Importantly, the viral modulation of host signaling pathways can change the activity of transcription factors downstream in the signaling cascade, resulting in an altered host gene transcriptome. In the case of SARS-CoV-2, it is still poorly understood which host signaling pathways are usurped by the virus, which can promote infection and viral pathogenesis. This study aimed to fill this knowledge gap by screening the effect of each of the SARS-CoV-2 proteins on 10 different host signaling pathways, many of which are known to be critical in the regulation of viral infections.

## 2. Materials and Methods

### 2.1. Cell Lines and Plasmids

Cell lines 293T, HEK293 (ATCC), and Vero E6 (ATCC) were maintained in DMEM medium supplemented with 10% FBS and penicillin/streptomycin (P/S). The luciferase reporter plasmids of signaling pathways were purchased from Addgene (Watertown, USA). These plasmids were the following: Wnt signaling (#12456; M50 Super 8x TOPFlash), p53 signaling (#16442; PG13-Luc WT p53 binding sites), TGF-beta signaling (#16495; SBE4-Luc), c-Myc signaling (#16564; pBV-Luc WT MBS1-4), Hypoxia signaling (#26731; HRE-luciferase), Hippo signaling (#34615; 8xGTIIC-luciferase), AP-1 signaling (#40342; 3xAP1pGL3), Notch signaling (#41726; 4xCSL-luciferase), OCT4/SOX2 (#69445; 6xO/S luc), and NF-kB signaling (#111216; 4xNF-kB Luc). The p21 promoter luciferase plasmid was made by cloning 2.4 kb of DNA sequence upstream of the transcription start site of p21 gene into pGL3 luciferase plasmid. The V5-p53 expression vector was purchased from Addgene (#22945). The vectors expressing C-terminally 2xStrep epitope-tagged SARS-CoV-2 proteins were purchased from Addgene and were used in a study by Gordon DE et al. [8].

### 2.2. Luciferase Reporter Assay

293T and HEK293 were transfected with the luciferase reporter plasmids, along with gene expression plasmids, using the transfection reagent polyethylenimine (PEI 25K) (Polysciences, Warrington, USA). At 48 h post-transfection, the cells were collected in a lysis buffer (0.5% Triton-×100 diluted in 1X DPBS). Cell lysates were mixed with ONE-Glo luciferase substrate (Promega, Madison, USA), and the luciferase activity was measured using a Promega GloMax-Multi Detection System. All luciferase assays were carried out in triplicate. The luciferase reporter data show the average of the three independent luciferase experiments. Significance (*p*-value) was determined through a two-tailed Student’s *t* test, and *p* < 0.05 was considered statistically significant.

### 2.3. SARS-CoV-2 Infection and Plaque Assay

V5-p53 was expressed in Vero E6 cells by using lentiviral transduction. Two days after lentiviral transduction, the cells were trypsinized, and 250,000 cells were seeded in each well of a 6-well dish. The next day, the cells were infected with the clinical isolate UF-1 of SARS-CoV-2 [16] at a multiplicity of infection of 0.01 for 1 h. Following the 1 h adsorption period, cells were rinsed with PBS and replaced with fresh cell culture media. After 72 h incubation at 37 °C and 5% CO_2_, samples were collected, and plaque assays were performed by preparing ten-fold serial dilutions of samples and plated in triplicate on monolayers of Vero E6 cells in 24-well plates. After a 1 h adsorption period, cell monolayers were rinsed with PBS and replaced with overlay media (EMEM with 2% FBS and 1% methocellulose). Following a 72 h incubation at 37 °C and 5% CO_2_, cells were fixed and stained in a crystal violet solution (1% crystal violet in 100% methanol). Following a 30 min inactivation timepoint, cell monolayers were rinsed with PBS and plaques were enumerated.

## 3. Results and Discussion

**Figure 1:**
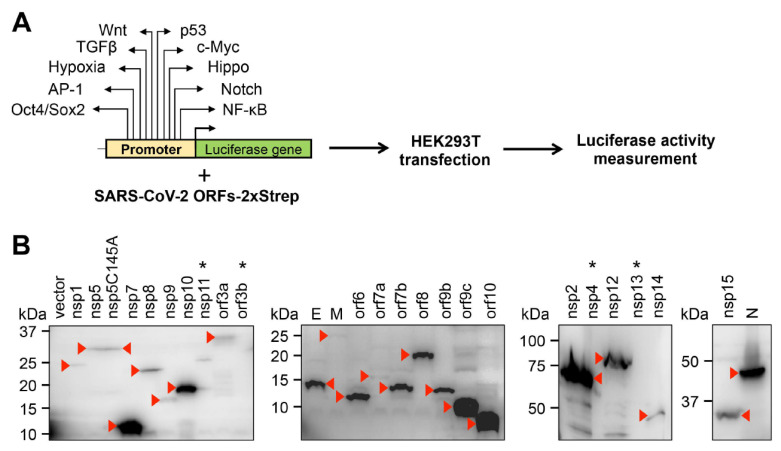
Experimental workflow for the study. (**A**) Outlining the signaling pathway luciferase reporter screen. The names of signaling pathways tested in this study are shown. (**B**) Immunoblot analysis of the expression of C-terminally 2xStrep-tagged SARS-CoV-2 proteins in transfected 293T cells using Strep-tag antibody. The red arrowheads indicate the viral proteins on the immunoblots. Viral proteins that are not expressed are indicated by an asterisk.

To investigate the effect of SARS-CoV-2 proteins on host signaling pathways, we performed a luciferase reporter screen utilizing reporter plasmids, in which the promoters of luciferase gene were responsive to distinct signaling pathways, such as Wnt, p53, TGFβ, c-Myc, Hypoxia, Hippo, AP-1, Notch, Oct4/Sox2, and NF-κB (Figure 1A). We co-transfected 293T cells with luciferase reporter plasmids, with vectors expressing 25 different SARS-CoV-2 proteins that were C-terminally 2xStrep epitope-tagged [8] and measured the luciferase activity 48 h after transfection. Figure 1B shows the immunoblot analysis of the expression of 25 viral proteins using a Strep-tag antibody. Note that we could not detect the expressions of four of the viral proteins (nsp4, nsp11, nsp13, orf3b), and thus we omitted these proteins from further experiments.

The results of the signaling pathway screen are summarized in Table 1, Tables S1 and S2. We used a 5-fold change in luciferase activity as a threshold to capture viral proteins that have the most robust effect on host signaling pathways. However, we did not exclude the possibility that viral proteins that induced less than a 5-fold change may also be biologically important, which requires further investigation. Based on our analysis, we identified five viral proteins that inhibited and 12 viral factors that could induce the reporter promoters of different signaling pathways (Table 1). Interestingly, we found only viral activators for Hypoxia, Notch, and NF-κB signaling pathways; one viral inhibitor for c-Myc; and both viral activators and repressors for p53, TGFβ, and AP-1 signaling pathways. In addition, we identified nsp5, nsp15, and orf6 only as repressors, while nsp9, nsp10, nsp14, E, M, N, orf7a, orf9a, orf9b, and orf10 acted only as inducers of different signaling pathway reporters. In contrast, the viral proteins nsp1 and orf3a worked either as activators or repressors of different signaling pathway reporters. Under our experimental conditions, we could not identify any SARS-CoV-2 proteins that have a robust effect on the Wnt, Hippo, and Oct4/Sox2 signaling pathway reporter promoters; in addition, we could not detect any effects on any of the signaling pathway reporters with six viral proteins (nsp2, nsp7, nsp8, nsp12, orf7b, and orf8). Interestingly, we found that ORF9b, ORF9c, and ORF10 can induce the reporter promoters of five different signaling pathways. ORF9b and ORF9c were shown to be able to suppress innate immunity and modulate mitochondria-associated functions and ER stress response, respectively [17,18]. The importance of ORF10 in SARS-CoV-2 infection is controversial as one study reported that ORF10 is dispensable for viral replication, while another study showed that ORF10 can promote viral replication by inhibiting the MAVS-mediated type I IFN signaling pathway [19,20]. It is unclear why these viral proteins can affect multiple distinct signaling pathway reporter promoters. We hypothesize that many of our hits in the screen may indirectly regulate signaling pathways by modulating intracellular conditions that affect the activity of signaling pathways. Because of the limited coding capacity of the SARS-CoV-2 RNA genome, it is not surprising that several viral proteins evolved to deregulate multiple signaling pathways, reprogram the infected cells, and protect them from antiviral immunity to support viral replication. Further investigations are warranted to ascertain the role of different SARS-CoV-2 proteins in the modulation of signaling pathways during viral infection.

Importantly, we found multiple viral proteins that have the potential to control the TGFβ, AP-1, and NF-κB host signaling pathways, which are major regulators of the immune response pathways and are dysregulated during SARS-CoV-2 infection in patients. In fact, a recent study showed that orf3a, M, and orf7a of SARS-CoV-2 are NF-κB activators, and orf7a-mediated NF-κB activation triggers an elevated expression of cytokines and chemokines, which are frequently increased in severely affected COVID-19 patients [21]. In agreement with this previous report, we also found that these viral proteins can strongly induce an NF-κB responsive promoter, and orf7a was notably the most potent NF-κB activator (Table 1). The TGFβ signaling pathway was also recently shown to be a major player in the induction of chronic immune response upon SARS-CoV-2 infection in COVID-19 patients requiring prolonged ICU care [22]. We identified several viral inducers of TGFβ reporter that could be involved in the SARS-CoV-2-induced TGFβ-mediated immunopathology, which remains to be shown by future studies (Table 1). Hypoxia has also been implicated in contributing to the development of cytokine storm in COVID-19 patients, a signaling pathway for which we could also identify several putative viral inducers (Table 1) [23]. Similarly, the role of the Notch pathway in promoting the pro-inflammatory microenvironment and cardiovascular diseases (e.g., myocarditis, thrombosis) that are associated with COVID-19 is well-established, but it is still unknown which SARS-CoV-2 factors are involved in the dysregulation of the Notch pathway [24,25]. We identified at least five viral inducers that could play a role in the regulation of the Notch signaling pathway in SARS-CoV-2-infected cells (Table 1). We note that many of the signaling pathways affected by SARS-CoV-2 proteins were also shown to play a role in the regulation of specific organ functions; thus, the dysregulation of these signaling pathways can also contribute to organ failure in COVID-19 patients [6,26].

**Figure 2:**
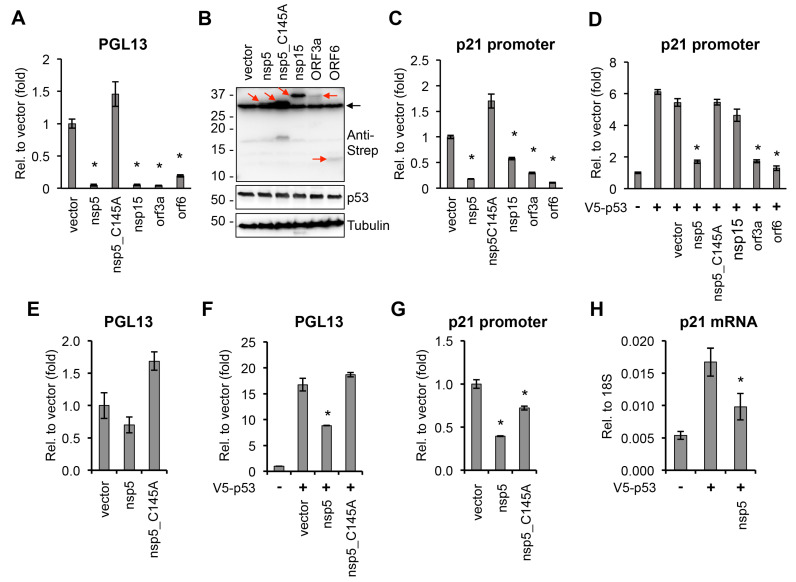
Effect of SARS-CoV-2 proteins on p53-regulated promoters. (**A**) PG13-Luc reporter plasmid, which includes p53 binding sites, was co-transfected with vectors expressing the indicated viral proteins into 293T cells. Luciferase activity was measured 48 h after transfection and is shown relative to the luciferase activity in the vector-transfected sample. (**B**) Immunoblot analysis of the expression of 2xStrep-tagged viral proteins and p53 in 293T cells. Red arrows point at the coronavirus proteins. Black arrow indicates non-specific band. (**C**) p21 promoter luciferase plasmid and SARS-CoV-2 factors were co-transfected into 293T cells and the luciferase activity was captured at 48 h post-transfection. (**D**) The same luciferase assay as described in panel C was performed, except the cells were also transfected with a V5-p53 expression plasmid. (**E**) The luciferase assay shown in panel A was repeated in HEK293 cells. (**F**) PG13-Luc reporter plasmid was co-transfected with nsp5 or its mutant into HEK293 cells, and luciferase activity was measured 48 h after transfection and is shown relative to the luciferase activity in the vector-transfected sample. (**G**) p21 promoter luciferase plasmid and nsp5 or its mutant were co-transfected into HEK293 cells, and the relative luciferase activity was measured at 48 h post-transfection. (**H**) RT-qPCR analysis of p21 gene expression in vector-, V5-p53-, and V5-p53/nsp5-2xStrep-transfected HEK293 cells. *t* test was performed between vector- and SARS-CoV-2 ORF-transfected samples (* *p* < 0.05).

The role of p53 in the regulation of the cell cycle, DNA repair, apoptosis, and cellular stress responses is well-characterized [27]. In addition, p53 can also be involved in regulating innate immune responses, such as stimulating type I IFN response, and it can have both positive and negative effects on viral infections [27,28,29]. Strikingly, we identified four inducers and four repressors among SARS-CoV-2 proteins for p53-mediated promoter activation, suggesting that the regulation of p53 activity could be crucial during SARS-CoV-2 infection (Table 1). We found that one of the most potent p53 repressors was the main viral protease nsp5. Interestingly, the protease mutant of nsp5 (C145A) did not inhibit the activity of the p53-regulated reporter promoter, indicating that the protease function of nsp5 is required for downregulating p53 transcriptional activity (Table 1) [8]. We confirmed the result of our luciferase reporter screen in an independent luciferase reporter assay that nsp5, nsp15, orf3a, and orf6 could significantly inhibit the activity of a p53-regulated promoter, while the C145A mutant of nsp5 could not (Figure 2A). Immunoblot analysis showed that the p53 expression level was not affected under our experimental conditions (Figure 2B). The transcriptional activity of p53 in these luciferase assays was measured by using the frequently employed p53 reporter plasmid PG13-Luc, in which a minimal promoter is fused to 13 copies of a p53 binding site [30]. In order to test the effects of the viral proteins (nsp5, nsp15, orf3a, and orf6) on a bona fide p53-regulated cellular promoter, we focused on the promoter of the cell cycle regulatory gene p21, which is a well-characterized target of p53 and possesses two p53 response elements (Figure 2C,D) [30]. We measured the effect of the coronavirus proteins both on the basal activity of the p21 promoter (Figure 2C) and on the p53-induced p21 promoter (Figure 2D) using a luciferase assay. Our results show that, while nsp5, nsp15, orf3a, and orf6 could inhibit the basal activity of the p21 promoter, all but nsp15 could repress the p53 overexpression-induced p21 promoter. In line with the luciferase assays using PG13-Luc, we found that the nsp5 mutant C145A could not exert its repressive effect on p53 at the p21 promoter either. Since 293T expresses the SV40 T antigen, which can inhibit p53 activity and may interfere with the nsp5-mediated suppression of p53, the reporter promoter luciferase assays were repeated in HEK293 cells (Figure 2E–G). We confirmed that nsp5 inhibits p53-mediated promoter activation and can downregulate the expression of p53-regulated p21 gene in cells (Figure 2E–H). These data support the notion that nsp5 inhibits p53-mediated promoter activation, which requires its protease function.

**Figure 3:**
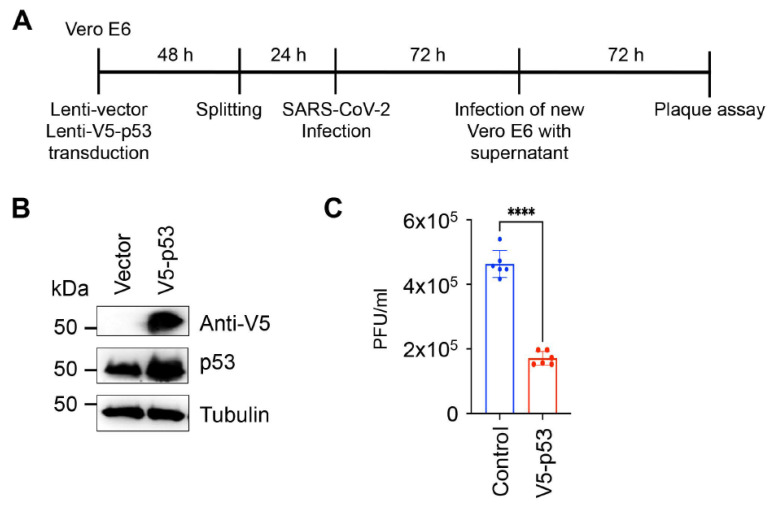
Inhibition of SARS-CoV-2 virus production by p53. (**A**) Experimental workflow of SARS-CoV-2 infection and measurement of virus production. (**B**) Immunoblot analysis of p53 expression in Vero E6 cells. (**C**) Vector control and V5-p53 were introduced into Vero E6 cells by lentiviral transduction. Two days later, the same number of cells was infected with the clinical isolate UF-1 of SARS-CoV-2 at a multiplicity of infection of 0.01 for 1 h. At 72 h post-infection, the supernatants were collected and used for the plaque assay on Vero E6 monolayers. Plaque assay was analyzed 72 h later. The data are based on the average of six independent SARS-CoV-2 infections of lentivirus-transduced cells. Dots represent the 6 replicates that were used for calculation. Significance was determined using a two-tailed unpaired *t*-test (**** *p* < 0.0001).

Previous studies have shown that p53 can have both antiviral and proviral activities [29,31]. To investigate the impact of p53 on SARS-CoV-2 production, we infected Vero E6 cells overexpressing V5 epitope-tagged p53 with the clinical isolate UF-1 of SARS-CoV-2 and measured virus production 72 h after infection (Figure 3A) [16]. We found that the overexpression of p53 significantly reduced virus production, indicating that p53 acts as a negative regulator of virus production in SARS-CoV-2-infected cells (Figure 3B,C). Our result is in agreement with previous studies, which demonstrated that p53 can inhibit the replication of coronaviruses, such as SARS-CoV and HCoV-NL63 [32]. It was demonstrated that the viral factor nsp3 of these coronaviruses interacts with the host E3 ubiquitin ligase RCHY1, which augments RCHY1-mediated ubiquitination and the degradation of p53. Given that the protease mutant nsp5 of SARS-CoV-2 could not repress p53-mediated promoter activation (Figure 2), it is possible that nsp5 can also directly degrade either p53 or a co-factor of p53 that is required for the transcriptional activity of p53. Although we did not detect p53 protein degradation by nsp5, a recent study found that p53 protein expression can be downregulated by nsp5 to some degree [33]. Interestingly, most of the host proteins whose abundance is decreased by nsp5 did not interact with nsp5, indicating that depletion of host proteins by nsp5 may be an indirect effect of nsp5 activity [33]. In fact, p53 has not yet been detected in any nsp5 interactomes so far. Nsp5-mediated p53 cleavage remains to be tested in future studies. Another mechanism by which coronaviruses can inhibit p53 activity has been described for orf6 of SARS-CoV, which blocks the nuclear import of p53, thereby hindering the transcriptional function of p53 [34]. In our screen, we also identified orf6 of SARS-CoV-2 as a potent repressor of the p53 pathway, which was also confirmed by using the p53-controlled p21 promoter (Table 1 and Figure 2). Whether SARS-CoV-2 orf6 works in a similar way to that of SARS-CoV remains to be determined. It is noteworthy that we could identify not only p53 inhibitors but also p53 activators among SARS-CoV-2 proteins. It is possible that SARS-CoV-2 can induce and repress p53 signaling depending on the cellular environment or the stage of the viral life cycle. In fact, SARS-CoV-2 proteins have been reported to be able to block or induce the type I IFN signaling pathway, resulting in an aberrant IFN response during viral infection [35].

In summary, we identified several coronavirus proteins that can control various cellular signaling pathways, which have not been explored in previous studies. Our results provide a foundation for future studies, which will determine how the coronavirus proteins that we identified in our screen can control distinct host signaling pathways during SARS-CoV-2 infection, and how these novel virus–host interactions can contribute to the progression of SARS-CoV-2 infection and COVID-19-associated multiple organ failure. One of our novel findings is the identification of p53 as a component of the host intrinsic antiviral immunity that restricts SARS-CoV-2 replication. Importantly, we found several viral proteins that can dysregulate the transcriptional activity of p53. Because p53 is a pleiotropic host factor, the modulation of p53 activity by SARS-CoV-2 impacts many different cellular processes that can contribute to the diverse pathology of COVID-19.

## Figures and Tables

**Table 1:** Summary of the results of the signaling pathway luciferase reporter screen. Data show the average of three independent luciferase assays (*n* = 3). Fold change relative to the vector-transfected sample and standard deviations are shown. Green ≥ 5-fold upregulation, red ≥ 5-fold downregulation.

		Host Signaling Pathways									
		Wnt	p53	TGFβ	c-Myc	Hypoxia	Hippo	AP-1	Notch	Oct4/Sox2	NFkB
**SARS-CoV-2 proteins**	**vector**	1.0 ± 0.1	1.0 ± 0.1	1.0 ± 0.4	1.0 ± 0.2	1.0 ± 0.03	1.0 ± 0.04	1.0 ± 0.1	1.0 ± 0.3	1.0 ± 0.1	1.0 ± 0.1
	**nsp1**	0.7 ± 0.1	2.2 ± 0.1	0.2 ± 0.02	0.1 ± 0.002	1.6 ± 0.2	0.5 ± 0.04	0.03 ± 0.002	5.6 ± 2.0	1.3 ± 0.1	11.1 ± 0.9
	**nsp2**	1.0 ± 0.2	0.8 ± 0.1	2.4 ± 0.5	2.0 ± 0.1	3.1 ± 0.5	0.9 ± 0.3	1.3 ± 0.03	2.3 ± 0.8	0.8 ± 0.1	1.7 ± 0.7
	**nsp5**	1.1 ± 0.3	0.05 ± 0.01	0.4 ± 0.1	0.5 ± 0.02	0.5 ± 0.1	0.4 ± 0.1	0.1 ± 0.001	1.3 ± 0.4	0.7 ± 0.1	0.7 ± 0.1
	**nsp5 C145A**	0.7 ± 0.2	1.5 ± 0.2	2.2 ± 0.4	1.4 ± 0.1	2.3 ± 0.2	0.8 ± 0.2	0.4 ± 0.1	1.9 ± 0.4	0.7 ± 0.1	1.2 ± 0.2
	**nsp7**	0.8 ± 0.1	0.7 ± 0.1	3.6 ± 0.5	1.7 ± 0.2	3.2 ± 0.3	1.0 ± 0.1	0.5 ± 0.1	1.1 ± 0.4	0.5 ± 0.03	1.0 ± 0.1
	**nsp8**	0.8 ± 0.2	1.0 ± 0.1	2.9 ± 0.3	1.4 ± 0.2	4.0 ± 0.6	0.8 ± 0.1	0.6 ± 0.02	1.5 ± 0.6	0.6 ± 0.04	0.8 ± 0.1
	**nsp9**	1.0 ± 0.1	0.9 ± 0.1	5.1 ± 0.4	1.6 ± 0.3	3.3 ± 0.3	1.1 ± 0.1	0.6 ± 0.1	2.0 ± 0.3	0.9 ± 0.1	1.6 ± 0.3
	**nsp10**	1.0 ± 0.1	0.3 ± 0.03	5.1 ± 0.7	2.0 ± 0.1	2.7 ± 0.8	1.0 ± 0.1	0.7 ± 0.1	2.2 ± 0.4	0.8 ± 0.04	1.5 ± 0.3
	**nsp12**	0.8 ± 0.05	0.9 ± 0.1	4.2 ± 0.7	3.1 ± 0.6	3.3 ± 0.8	0.8 ± 0.04	0.7 ± 0.01	3.3 ± 0.5	0.7 ± 0.1	2.0 ± 0.3
	**nsp14**	1.4 ± 0.3	2.3 ± 0.3	2.6 ± 0.4	1.9 ± 0.2	5.4 ± 0.3	3.3 ± 0.3	3.7 ± 0.1	17.7 ± 3.2	2.6 ± 0.1	12.6 ± 0.3
	**nsp15**	0.8 ± 0.1	0.1 ± 0.01	1.2 ± 0.2	1.0 ± 0.01	2.3 ± 0.2	0.6 ± 0.01	0.2 ± 0.03	1.4 ± 0.2	0.5 ± 0.1	1.5 ± 0.1
	**E**	1.1 ± 0.1	0.4 ± 0.1	4.4 ± 0.1	0.7 ± 0.1	3.1 ± 0.5	0.7 ± 0.04	0. ± 0.2	2.7 ± 0.6	1.0 ± 0.01	10.4 ± 1.5
	**M**	0.9 ± 0.1	0.5 ± 0.02	1.0 ± 0.3	0.6 ± 0.1	2.5 ± 0.1	0.3 ± 0.03	0.3 ± 0.1	1.8 ± 0.2	0.8 ± 0.1	7.3 ± 0.8
	**N**	1.6 ± 0.4	11.7 ± 1.2	6.3 ± 0.4	3.1 ± 0.4	4.4 ± 0.5	1.5 ± 0.2	1.4 ± 0.3	4.6 ± 0.3	0.9 ± 0.1	3.9 ± 0.6
	**orf3a**	0.8 ± 0.1	0.04 ± 0.01	1.1 ± 0.2	0.6 ± 0.1	1.8 ± 0.1	0.4 ± 0.1	0.4 ± 0.02	1.6 ± 0.6	0.6 ± 0.02	11.1 ± 0.9
	**orf6**	0.8 ± 0.2	0.2 ± 0.02	0.2 ± 0.01	0.3 ± 0.1	0.8 ± 0.2	0.7 ± 0.1	0.1 ± 0.02	1.4 ± 0.3	0.8 ± 0.1	0.9 ± 0.1
	**orf7a**	1.2 ± 0.1	1.7 ± 0.1	2.8 ± 0.1	1.2 ± 0.1	5.4 ± 0.8	0.8 ± 0.1	3.3 ± 0.4	3.1 ± 0.2	0.7 ± 0.1	59.2 ± 7.1
	**orf7b**	1.0 ± 0.3	0.4 ± 0.03	0.3 ± 0.01	0.4 ± 0.04	2.9 ± 0.5	0.7 ± 0.05	0.4 ± 0.1	2.5 ± 0.9	0.7 ± 0.1	3.5 ± 0.8
	**orf8**	0.8 ± 0.1	2.1 ± 0.04	1.6 ± 0.3	0.8 ± 0.1	2.5 ± 0.3	0.8 ± 0.1	0.4 ± 0.1	1.7 ± 0.4	0.6 ± 0.1	2.1 ± 0.2
	**orf9b**	4.8 ± 0.7	19.9 ± 2.2	16.0 ± 1.4	2.4 ± 0.4	20.8 ± 0.5	4.0 ± 0.4	15.4 ± 1.0	13.3 ± 1.8	2.9 ± 0.3	10.6 ± 0.8
	**orf9c**	3.5 ± 0.1	8.3 ± 0.4	14.5 ± 2.0	2.2 ± 0.3	13.7 ± 1.0	2.9 ± 0.1	3.9 ± 0.6	11.1 ± 0.9	1.8 ± 0.5	10.0 ± 0.5
	**orf10**	3.6 ± 0.3	9.9 ± 0.4	14.9 ± 1.2	2.2 ± 0.1	15.6 ± 0.4	3.0 ± 0.1	5.0 ± 0.5	11.1 ± 2.7	2.0 ± 0.1	12.3 ± 0.7

## Supplementary Materials

The following supporting information can be downloaded at: https://www.mdpi.com/article/10.3390/v14051039/s1, Table S1: Summary of the raw luciferase measurement values. Boxes in green and red indicate positive hits in the screen, which are also shown in Table 1. Table S2: Summary of the statistical analysis of the signaling pathway luciferase reporter screen. The *p* values were determined by two-tailed Student’s *t* test. Boxes in green and red indicate positive hits in the screen, which are also shown in Table 1.
