# Supplementary material for: Signaling Pathway Reporter Screen with SARS-CoV-2 Proteins Identifies nsp5 as a Repressor of p53 Activity
Source: Viruses. 2022 May 13;14(5):1039. doi: 10.3390/v14051039 (PMC9145535; doi:10.3390/v14051039)
Supplement: Supplementary file 1 [file viruses-14-01039-s001.zip › Table S1 legend.pdf]

Table S1. Summary of the raw luciferase measurement values. Boxes in green and red indicate positive hits in the screen, which are shown in Table 1.
