# Supplementary figures and images for: Signaling Pathway Reporter Screen with SARS-CoV-2 Proteins Identifies nsp5 as a Repressor of p53 Activity
Source: Viruses. 2022 May 13;14(5):1039. doi: 10.3390/v14051039 (PMC9145535; doi:10.3390/v14051039)

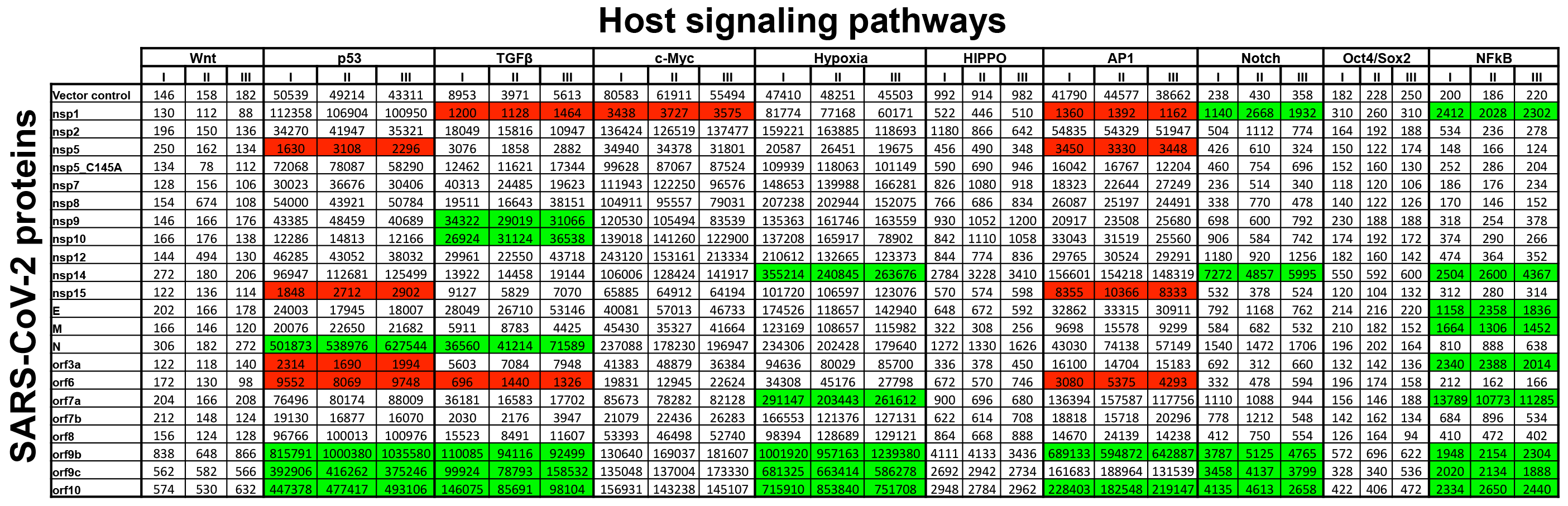

Supplement: Supplementary file 1 [file viruses-14-01039-s001.zip › Table S1.tif]

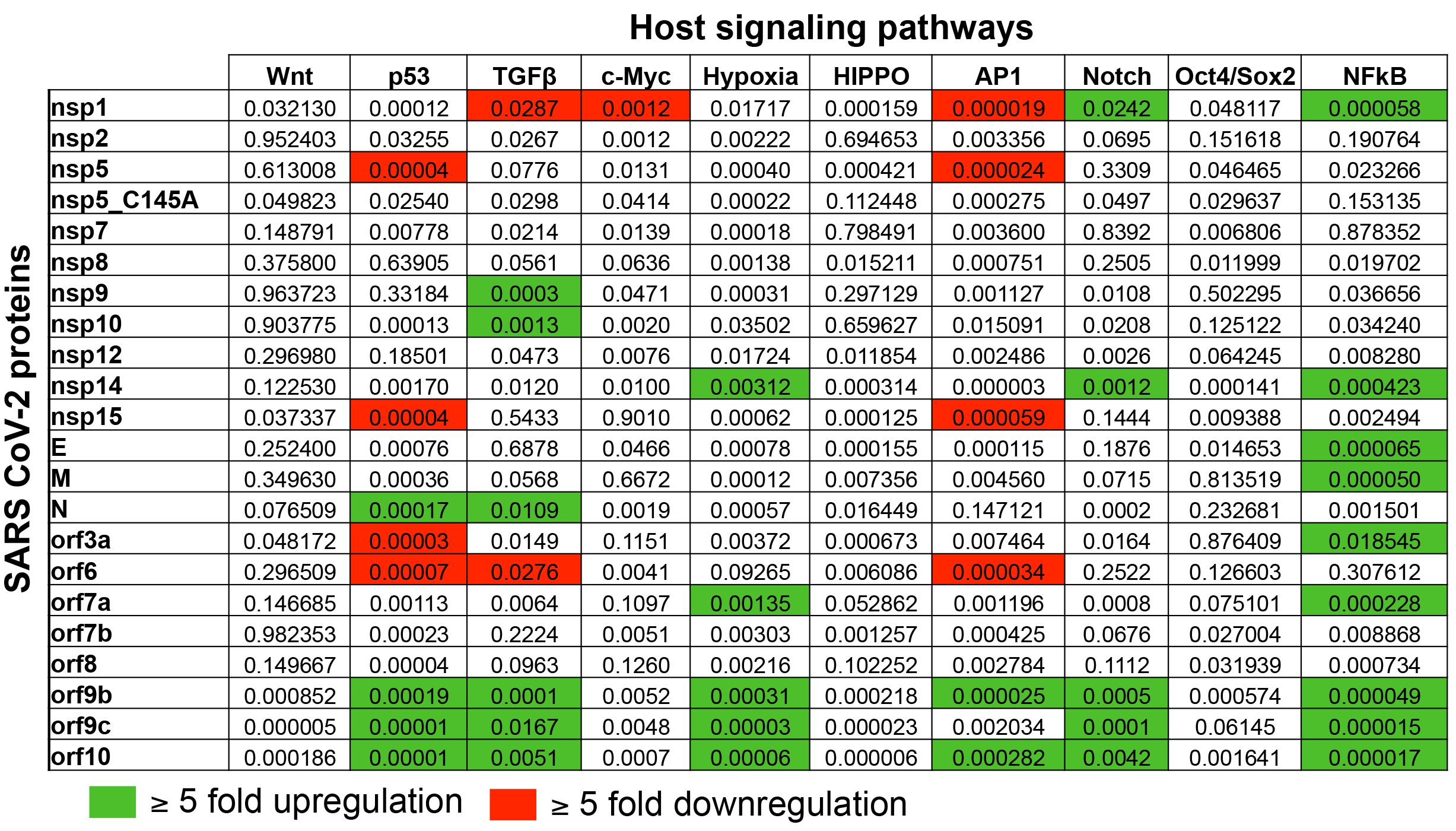

Supplement: Supplementary file 1 [file viruses-14-01039-s001.zip › Table S2.tif]
